# The Potential of Phylogenetically Diverse Culturable Actinobacteria from *Litopenaeus vannamei* Pond Sediment as Extracellular Proteolytic and Lipolytic Enzyme Producers

**DOI:** 10.21315/tlsr2022.33.3.10

**Published:** 2022-09-30

**Authors:** Diah Ayuningrum, Aninditia Sabdaningsih, Oktavianto Eko Jati

**Affiliations:** 1 Department of Aquatic Resources, Faculty of Fisheries and Marine Sciences, Universitas Diponegoro, Semarang 50241, Indonesia; 2 Tropical Marine Biotechnology Laboratory, Faculty of Fisheries and Marine Sciences, Universitas Diponegoro, Semarang 50241, Indonesia

**Keywords:** *Streptomyces*, *Nocardiopsis*, Bioremediation, Organic Waste, 16S rDNA

## Abstract

Enzymes are catalysts that can increase the reaction time of a biochemical process. Hydrolytic enzymes have a pivotal role in degrading organic waste in both terrestrial and aquatic environments. The aims of this study were (1) to investigate the ability of actinobacteria isolated from *Litopenaeus vannamei* pond sediment to produce proteolytic and lipolytic enzymes, (2) to identify promising candidates using 16S rRNA gene amplification, and (3) to construct a phylogenetic tree based on the 16S rRNA genes. A skim milk agar medium was used in the preliminary experiment of the proteolytic assay, and a Tween 20/80 medium was used in the lipolytic assay. Fifteen and 20 (out of 40) actinobacterial isolates showed great potential for proteolytic and lipolytic activities, respectively. Furthermore, four actinobacteria isolates produced both enzyme types with proteolytic and lipolytic index scores of 1–6.5. The most promising candidates were SA 2.2 (IM8), SC 2.1 (IM6), SD 1.5 (IM6) and SE 1.1 (IM8). BLAST homology results showed a high similarity between the actinobacteria isolates and *Streptomyces verucosisporus, S. mangrovicola, S. barkulensis* and *Nocardiopsis lucentensis*, respectively. Therefore, actinobacteria from *Litopenaeus vannamei* pond sediment are high-potential proteolytic and lipolytic enzyme producers.

**Highlights**
- Actinobacteria isolates from *Litopennaeus vannamei* sediments are capable of producing extracelluler proteolytic and lipolytic enzymes.
- BLAST homology resulted in four most promising enzyme produces from genera Streptomyces and Nocardiopsis.
- Genus Streptomyces dominated both proteolytic and lipolytic enzymes production from *L. vannamei* pond environment.

## INTRODUCTION

Enzymes are catalysts that act under standard conditions and can promote chemical reactions with remarkable efficiency and specificity. The use of hydrolytic enzyme such as protease, amylase, xylanase and chitinase in industrial as well as medical field has increasing (Manisha & Yadav 2017). Indonesian estimated the consumption of 2,500 tons of enzymes for its industries with around IDR200 billion import value in 2017, and of 5%–7% average volume growth rate per year (Hamdani *et al*. 2019). These values are significant enough to encourage Indonesia’s efforts for independent enzyme production by started looking for new sources of enzymes producers.

Proteases are hydrolytic enzyme that breaks down proteins into simpler molecules when in contact with water. Protease is essential for all living beings because it drives the process of protein metabolism, and aids in the digestion of proteins in food and the reuse of intracellular proteins such as enzymes, hormones and neurotransmitters. Inside the cells, proteases function within the cell membrane border, i.e., degeneration of unused enzymes, recycle of misfolded proteins and other housekeeping tasks in the cell (Krishnappa *et al*. 2014). Another kind of hydrolytic enzyme is lipase, which consist of unique and diverse type that catalyse the breakdown of fats and oils through the hydrolysis process. As well as protease, lipases also have biocatalysts potential in biotechnology process.

Many plants, animals and even microorganisms are able to produce both protease and lipase enzymes. However, protease from microbes are more preferred than from plants and animals, because microbes is produce higher protease with more than 60% of global consumption (Beg & Gupta 2003). Furthermore, microbial proteases are more applicable for industry rather than animal and plant proteases because of the presence of unique characteristics (Palsaniya *et al*. 2012; Sathishkumar *et al*. 2015). Thus, making the microbial protease has higher value and successfully dominant the global enzyme market (Gupta *et al*. 2002; Gupta & Ramnani 2006; Vijayaraghavan *et al*. 2014). Global enzyme markets account more than 60% protease which being used in bio-process industry such as textile, leather, meat tenderisation, cheese, baking, brewery, dehairing, organic synthesis, pharmaceuticals as well as wastewater treatments (Sun *et al*. 2019; Pastor *et al*. 2001). Recently, beside microbial protease, microbial lipase also attracted attention due to the biotechnological potential in industry such as laundry detergents as well as stereospecific biocatalysis similar to microbial protease (Jaeger *et al*. 1994; Jaeger & Reetz 1998). The producers of this extracellular microbial lipase are mostly bacteria and fungi (Chandra *et al*. 2020). Lipase enzyme originated from bacteria and fungi are different from one another in the physical and biochemical features, thus having specific capabilities in catalysing the enzymatic reaction. In general, all type of lipase has become one of the leading biocatalysts with approximate multibillion-dollar contribution to the industry.

One extensively studied protease and lipase producer is actinobacteria. Actinobacteria is a large group of mostly Gram-positive bacteria with high content of G+C and secrete many primary and secondary metabolites. The primary metabolites include extracellular enzymes that have a wide range of biological roles, and some of them involve in organic compounds degradation (Mukhtar *et al*. 2017). Actinobacteria are widely distributed both in terrestrial as well as in aquatic ecosystems. In aquatic ecosystems, actinobacteria is commonly found in the sediments, since actinobacteria is commonly inhabitant in the soil. This distribution allows them to contribute to many important ecological processes, i.e., biogeochemical cycle and bioremediation of ecosystems (Chen *et al*. 2015).

*Litopennaeus vannamei* ponds are one of aquatic ecosystem with high organic waste production that can damage the surrounding environment if not handled properly. This organic waste come from the accumulation from feed residue, feces, shrimp carapaces, dead plankton, as well as the metabolite waste in the bottom of the ponds (Suwoyo *et al*. 2015). The natural decomposition of this organic wastes release toxin to the pond and causes diseases in vannamei shrimp (Patang 2016). Therefore, management of microbial ecology is needed to optimise biogeochemical as well as carbon cycle in order to reduce sludge accumulation in *vannamei* ponds. Some members of the actinobacteria from genera *Streptomyces* and *Nocardiopsis* isolated from marine environment have been reported for their ability to produce hydrolytic enzymes such as lipase, amylase, chitinase, pectinase and ligninase enzymes, which have shown bioremediation potential in shrimp culture by significantly by reducing Biochemical Oxygen Demand (BOD) levels (Babu *et al*. 2018). Thus, the exploration of actinobacteria which able to produce hydrolytic enzymes from vannamei pond is needed. The goals of this study were to examine the qualitative activity of protease and lipase from actinobacteria isolated from *L. vannamei* pond sediment, to identify promising isolate candidates, and to construct the phylogenetic tree among the potential isolates.

## MATERIALS AND METHODS

### Isolation of the Actinobacteria

The sediment samples were collected from *L. vannamei* ponds in June 2020. Pond sediments were choosing because it contains a lot of organic waste from the feed residue, feces, shrimp carapaces and plankton which accumulated in the bottom of the pond. Therefore, we estimated that many actinobacteria producing hydrolytic enzymes were living in the *L. vannamei* pond sediments. The isolation of actinobacteria began by dilluting the sediment sample as many as 1 mL to 10 mL sterile seawater (1:10 v/v), then shaken vigorously using a shaker with 120 rpm for 1 min, settled 5 min, and inoculated on to the desired media as many as 50 μL (Ayuningrum *et al*. 2021). Two kinds of selective media employed to inoculate the actinobacteria were IM6 and IM8 media with the composition according to Bredholt *et al*. (2008). Both media were supplemented with antibiotic nalidixic acid (30 μg/mL) as anti-Gram negative bacteria and nystatin (70 μg/mL) as antifungal. The purpose of having two different media as isolation media was to increase the diversity of actinobacteria isolated from the pond sediment samples. The plates were then incubated at room temperature (25 ± 2°C) for 2 to 6 weeks. The grown actinobacterial colony with different morphological appearance were separated and purified to generate the axenic culture and stored in −80°C for further examination in the future.

### Reculture of The Actinobacteria

A total of 40 axenic actinobacteria cultures were recultured from storage using IM6 and IM8 selective media. The isolates from the cryotube were inoculated onto each medium using a streak plate technique. The incubation of the plates was at room temperature (28 ± 2°C) for 7 days and until the aerial hyphae started showing.

### Qualitative Proteolytic Activity Assay

In this study, we used skimmed milk agar (SMA) plate assay with the following composition for 1L volume: skim milk powder 2.8%, casein 0.5%, yeast extract 0.25%, dextrose 0.1% and agar 1.5% (Tennalli *et al*. 2012). Due to the protein content of skim milk powder, the SMA medium was sterilised in two steps as follows:

1. Skim milk powder was mixed with 25% of total distilled water (per 1 L) and sterilised at 105°C for 5 min.
2. An unmixed composition was sterilised at 121°C for 20 min.

Both solutions were then combined and poured onto a sterile petri dish. The actinobacteria cells from the colony were inoculated by forming a dot (small circle of the actinobacterial colony with d ≤ 2 mm) onto it, and two replicates were incubated for 2 weeks at room temperature (28 ± 2°C). The proteolytic activity of the isolates was detected as the formation of a holo zone surrounding the colony. Observations of the halo zone were made until days 14. The diameter of the halo zones and the colony themselves were measured three times with a vernier caliper. The proteolytic index (IP) was calculated according to Durham *et al*. (1987), namely as follows:


$$\text{IP}=\frac{\text{Diameter of clear zone }(\text{mm})-\text{Colony diameter }(\text{mm})}{\text{Colony diameter }(\text{mm})}$$

The IP score then divided become three categories as follows: high = IP ≥ 3.5; moderate = IP ≥ 1.5 < 3.5; and low = IP < 1.5. All of isolates showing proteolytic activity was further studied for its morphological characterisation. Three isolates with moderate to high IP score was identified using molecular approach to determine the closest species.

### Qualitative Lipolytic Assay

Testing for the potential of actinobacteria for lipolytic activity was conducted by precipitation testing using mixture of Tween 20/80 agar (Ramnath *et al*. 2017) with composition for 1 L as follows: agar 20 g, peptone 10 g, CaCl_2_._2_H_2_O 0.1 g, NaCl 5 g, mixture of Tween 20/80 10 mL (1: 1, v/v). Actinobacteria isolates were inoculated onto plates and incubated at 28 ± 2°C for 2 weeks in two replications. Isolates with lipolytic activity will have visible precipitation of calcium salt surrounding the colony. Observations of the precipitation zone were made until days 14. The precipitation zone diameter and the colony itself were measured three times with a vernier caliper. White precipitation around the colony boundary indicated lipase activity. The lipase activity index (LAI) was determined according to Willerding *et al*. (2011), namely as follows:


$$\text{LAI}=\frac{\text{Diameter of halo zone }(\text{mm})-\text{Diameter of the colony }(\text{mm})}{\text{Diameter of the Colony }(\text{mm})}$$

The LAI score then divided become three categories as follows: high = LAI ≥ 3.5; moderate = LAI ≥ 1.5 < 3.5; and low = LAI < 1.5. All of isolates showing lipolytic activity was further studied for its morphological characterisation. Three isolates with moderate to high LAI were further examined for their molecular features using 16S rRNA gene.

### Morphological Characterisation of Actinobacterial Isolates

The colony growing from the media were observed for the morphological appearance such as the shape, margin, elevation, surface, aerial hyphae and substrate hyphae. The morphological characterisation of the colony was done and published elsewhere in Ayuningrum *et al*. (2021). All of the isolates which showing either proteolytic or lipolytic activity were being observed microscopically. The microscopic characterisation of the colony was conducted using slide technique (Armaida & Khotimah 2016). Each media as well as IM6 and IM8 were cut into size of 1 cm × 1 cm using sterile scalpel and put on to the sterile object glass which previously placed inside the sterile petri dish. The spore from each isolate were put on to it, then covered with the cover glass and the petri dish were closed. Those plates were incubated in room temperature (25 ± 2°C) for 7 days. After 7 days, the cover glass with grown actinobacterial isolates were dripped with methylene blue and observed under microscope with magnification 10 × 100. The resulted images of actinobacteria were identified according to the Bergey’s Manual of Systematic Bacteriology (Goodfellow *et al*. 2012).

### Extraction of Genomic DNA and PCR of 16S rRNA Gene

The identification of the potential isolates was using the molecular approach, using 16S rRNA gene amplification. The most potential isolates were grown on the respective media (either IM6 or IM8) for seven days or until the aerial hyphae/spore was shown under room temperature incubation. After seven days, the aerial hyphae/spore were harvested and put into the microtube 1.5 mL to extract the genomic DNA. This DNA extraction was using a kit from Zymo Research, California (Quick-DNA Miniprep Plus Kit Catalog No. D4068 & D4069), following the biological fluids and cells protocol. After obtaining the DNA, the purity was measured with a Nanodrop Spectrophotometer (Thermo-Scientific Nanodrop 2000 UV-Vis) under wavelength A260/280. The DNA purity value between a range of 1.8–2.0 will be continued for the PCR process, other than that DNA purity value, the DNA extraction process was repeated. The amplification of partial 16S rRNA gene was amplified using polymerase chain reaction method (PCR) with universal primers pair:

1. PA 5′-AGA GTT TGA TCC TGG CTC AG-3′ and PH 5′-AAG GAG GTG ATCCAG CCG CA-3′ (Ayuningrum *et al*. 2019)
2. 27F 5′AGAGTTTGATCMTGGCTCAG-3 and 1492 R5′GGTTACCTTGTTACGACTT-3′ (Weisburg *et al*. 1991)

The final PCR reaction volume was 25 μL consisted of a 1 μL DNA template, 1 μL of each primer (forward and reverse), 9.5 μL of ddH_2_O, and a PCR mix (MyTaqTM Red Mix-Bioline) of 12.5 μL. The PCR reaction took place in a Thermal Cycler T100 (BIO-RAD), with process consisting of initial denaturation at 95°C for 10 min, then 34 chain cylcles of denaturation at 95°C for 45 s – annealing at 55°C for 60 s –extension at 72°C for 90 s, and a final extension at 72°C for 5 min. The PCRproducts were examined using gel electrophoresis (BIO-RAD) with agarose 1% for30 min at 100 volts. The gel was visualised with UVIDoc HD5 (UVITEC Cambridge, UK) to observe the PCR product. The PCR products with size of 1.500 bp weresent for sequencing to 1st base in Malaysia through Genetika Science, Indonesia.

### Analysis of BLAST Homology and Phylogenetic Tree

Both sequences resulting from the forward and reverse primers were aligned using MUSCLE in MEGA X program to create a consensus sequence (Kumar *et al*. 2018). The consensus sequence was processed with BLAST homology (Pearson 2013) in NCBI to determine the closest related species. The consensus results of the actinobacteria isolates were submitted to GenBank (http://www.ddbj.nig.ac.jp) to register the accession number. The sequences of five most potential isolates and other comparative species were aligned to generate a phylogenetic tree using statistical method Minimum Evolutionary Tree with the Tamura-Nei model and Bootstrap 1000 (Nei & Kumar 2000).

## RESULTS

### Qualitative Proteolytic Activity

**Figure 1:**
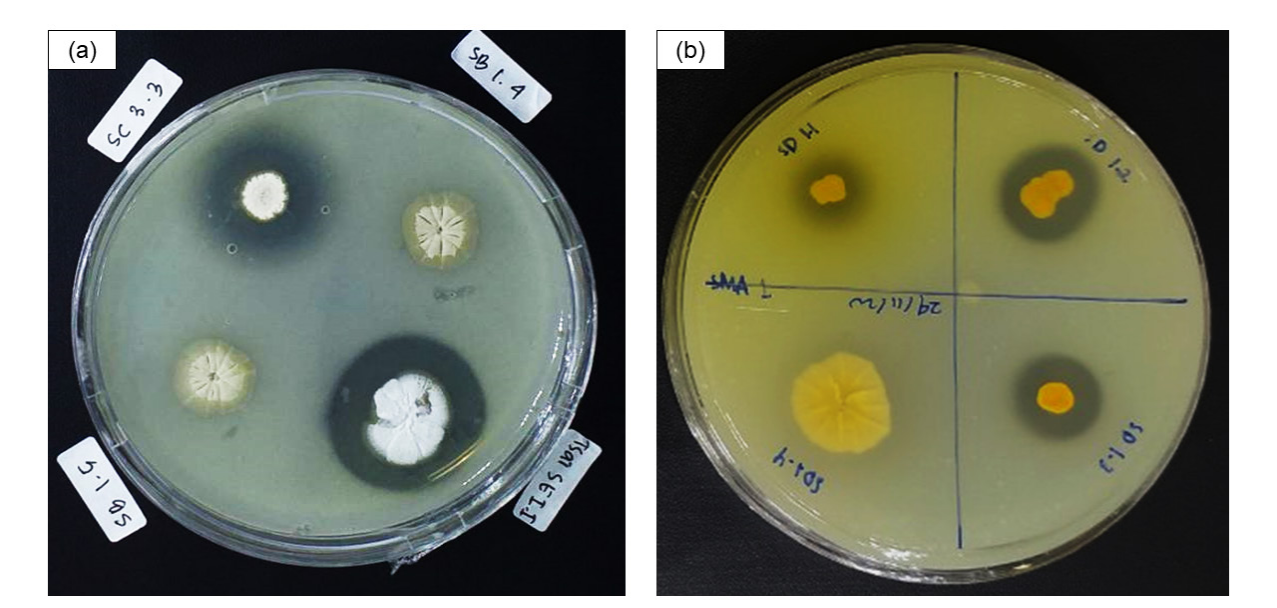
Qualitative proteolytic assay was conducted using skim milk powder as substrate in the medium. Extracellular microbial protease releasing by the actinobacterial isolates forming the halo zone surrounding the colony. The halo/clear zone can be seen (a) surrounds the isolate code SC 3.3, SE 1.1 and (b) surrounds isolate SD 1.1, SD 1.2 and SD 1.3.

The SMA-medium formed a halo zone surrounding the colony, as shown in Fig 1. The halo zone indicates that actinobacterial isolates catalysed the hydrolysisof peptide bond in SMA, resulting in a clear medium. The difference between isolates which produce extracellular protease and not, were clearly described in Fig 1. Isolates SC 3.3 (IM8), SE 1.1 (IM8), SD 1.1 (IM8), SD 1.2 (IM8) and SD 1.3 (IM8) were producing extracellular protease, meanwhile SB 1.5 (IM6), SB 1.4 (IM6) and SD 1.4 (IM8) were not producing the enzyme (no halo zone formed). The detailed results of the qualitative proteolytic assay for all isolates were shown in Table 1. Fifteen out of 40 actinobacterial isolates (37.5%) showed proteolytic activity. The higher Index of Protease (IP) indicates higher protease production from the actinobacterial isolates. According to Table 1, the highest IP value was from isolate SC 2.3 (IM6), followed by isolate SA 3.1 (IM6) and SD 1.5 (IM6) with each IP score of 4.30, 4.00 and 3.50, respectively. The IP score above 1.5 indicates that the isolates have good capability to produce protease than the other isolates. The isolates with IP lower than 1.5 were SB 4.1 (IM6), TGSE 4 (IM8) and SC 3.1 (IM8) with IP value of 1.2, 1.31 and 1.4, respectively. Other nine isolates showed medium proteolytic activity were isolates SA 2.7 (IM8), SC 2.1 (IM6), SC 3.3 (IM8), SC 3.4 (IM8), SD 1.1 (IM8), SD 1.2 (IM8), SD 1.3 (IM8), SE 1.1 (IM8), and SE 1.3 (IM8) with variety of IP ranging from 1.6–3.13.

### Qualitative Lipolytic activity

**Figure 2:**
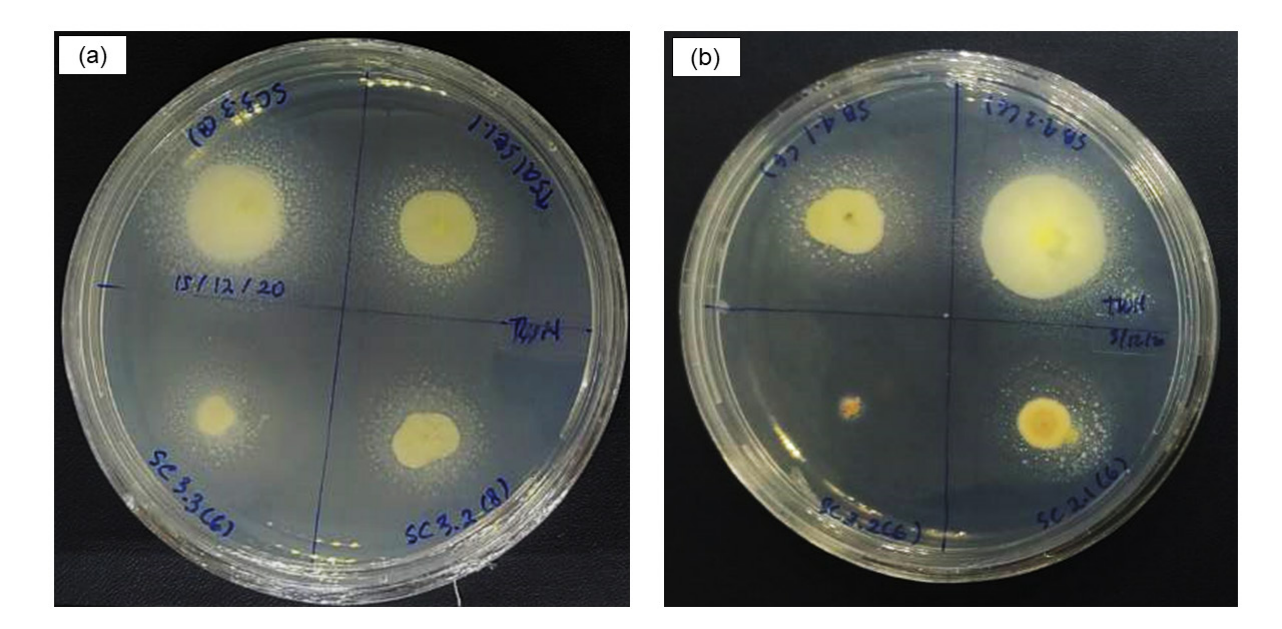
Qualitative lipolytic activity assay was conducted using Tween 20/80 agar medium. Extracellular microbial lypase releasing by the actinobacterial isolates forming the precipitation of calsium-salt surround the colony. From (a) all of the isolates was showning lypolityc activity, meanwhile (b) only one isolates (SC 3.2) not showing lipolytic activity.

The positive result is based on the precipitation principle, the hydrolysis of Tween by microbial lipase producing calcium salt as the fatty acid released. A total of 20 out of 40 isolates showed the lipolytic activity. The precipitation of calcium salt by the actinobacterial isolates was shown in Fig. 2. According to Fig 2, from eight isolates, only one isolate (SC 3.2 IM6) not showing any lipase activity. The other isolates of SC 3.2 (IM8), SC 3.3 (IM6), SC 3.3 (IM8), SE 1.1 (IM8), SB 4.1 (IM6), SB 4.2 IM6) and SC 2.1 (IM6) showed lipase activity indicated by the formation of precipitation zone. The precipitation zone was stated by the value of LAI in Table 1. The higher LAI indicates more lipase was produced by the actinobacterial isolates.

The highest LAI was from isolate SA 2.2 (IM8) followed by SB 1.2 (IM8) with the score 6.5 and 6.4, respectively. While the LAI score lower than 1.5 was found in isolates SA 2.2 (IM8), SA 2.4 (IM8), SA 2.5 (IM8) and SB 2.4 (IM8). The rest of the isolates (14 isolates) have moderate lipolytic activity indicated by the LAI score ranging from 1.5–3.2.

Based on the result of both proteolytic and lipolytic assay, we choose four most potential isolates to be identified morphologically (including microscopic observation) and molecularly. Those four candidates were isolates SA 2.2 (IM8), SC 2.1 (IM6), SD 1.5 (IM6) and SE 1.1 (IM8). The isolate SA 2.2 (IM8) has the highest LAI score among all of the actinobacterial isolates with the score 6.5. The other isolate SC 2.1 (IM6) and SE 1.1 (IM8) have both proteolytic and lipolytic activity with IP 2.22, 1.75 and LAI 2.4, 1.75, respectively. The last potential candidate SD 1.5 (IM6) was having high proteolytic activity with IP 3.5.

### Morphological Characterisation with Microscopic Approach

**Figure 3:**
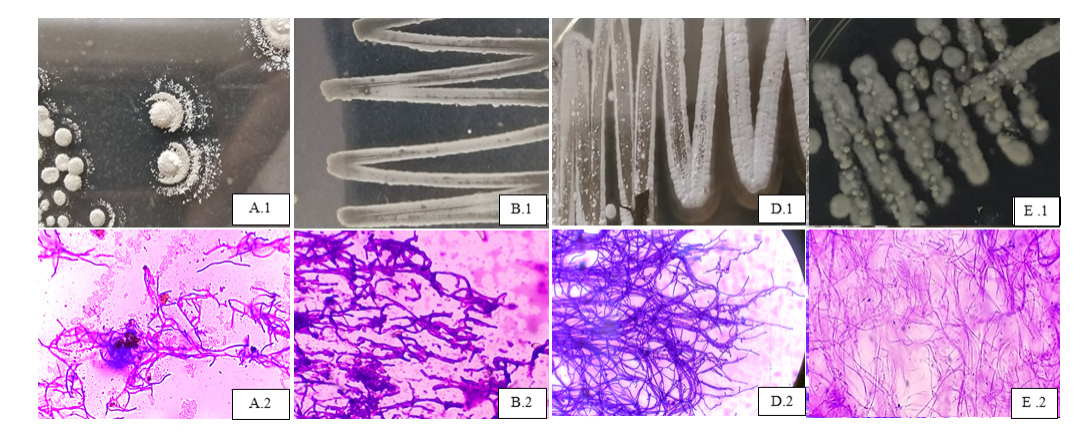
The morphological colony and microscopic observation of each potential isolates in producing extracelluler proteolytic and lipolytic enzymes. The isolates codes were A.1 and A.2: SA 2.2 (IM8), B.1 and B.2:SC 2.1 (IM6), C.1 and C.2: SD 1.5 (IM6), and D.1 and D.2: SE 1.1 (IM8).

The result of morphological characterisation of five most potential candidates were shown in Fig 3. According to Figs. 3A.1 and 3A.2, isolate SA 2.2 (IM8) has several character such as aerobic, powdery surface, white colour of aerial hyphae and spore, and big colony size. Furthermore, the microscopic observation of this isolate resulted in the extremely branching hyphae (both substrate and aerial), aerial hyphae form chains of spores before mature, and while mature much of single spores spread widely, the spore have oval-like shape. Isolate SC 2.1 (IM6) has the morphological character as follows: aerobic, powdery surface, yellow colour of substrate hyphae and light gray color of aerial hyphae (Fig. 3B.1). Microscopic observation of this isolate resulted in branching substrat and aerial hyphae, no septae found, has oval-shaped spore. The next potential isolate showed in Figs. 3C.1 and 3C.2 was SD 1.5 (IM6), which has morphological character as follows: aerobic, powdery surface, arial hyphae was white and substrate hyphae was slightly yellow, has spore with white colour, and has small colony size. The microscopic observation resulted in branching hyphae (both substrate and aerial), have cain-like structure at the tip of aerial hyphae, no septae found, have much spores. From the explanation before, three isolates of SA 2.2 (IM8), SC 2.1 (IM6) and SD 1.5 (IM6), typically have many similarities both from the colony morphology as well as the microscopic observation. All three isolates has powdery surface and spores with branching hyphae and no septae found. The last potential isolates seen in Figs. 3D.1 and 3D.2 was SE 1.1 (IM8). SE 1.1 was the only isolate that has different morphology than the other aformentioned three isolates before. This isolate has white velvety surface, aerobic, and no spores observed in the colony. Furthermore, the microcopic characterisation reveal the dense, thin, slim, smooth and branching substrate hyphae, no chain of spores observed at the tip of each hyphae, and the hyphae tip was pointy. Based on the morphological characterisation, we assumed that those five potential isolates belonged to two different group of actinobacteria. Furthermore, the one group of it was *Streptomycetaceae* and another was *Nocardiopsaceae*. The rest morphological characterisation of all active isolates can be seen in the Appendix.

### Molecular Identification with Phylogenetic Analysis

The BLAST result of consensus sequences from four potential isolates is shown in Table 2. In accordance with morphological characterisation, four isolates belonged to the genus *Streptomyces* and one isolate similar to the genus *Nocardiopsis*. The isolate SA 2.2 (IM8) showed 99.36% similarity with the species *Streptomyces verucosisporus*. The other isolate SC 2.1 (IM6) have similarity to the species *S. mangrovicola* with per ident 99.15%. Further, one more isolate which belonged to the genus *Streptomyces* was SD 1.5 (IM6) with per ident 99.21% with *S. barkulensis*. Finally, isolate SE 1.1 (IM8) was the only one isolate which belonged to the species *Nocardiopsis lucetens* with per ident 99.23%. All of the candidates were put into the phylogenetic tree to estimate the relationship with other closest species.

**Figure 5:**
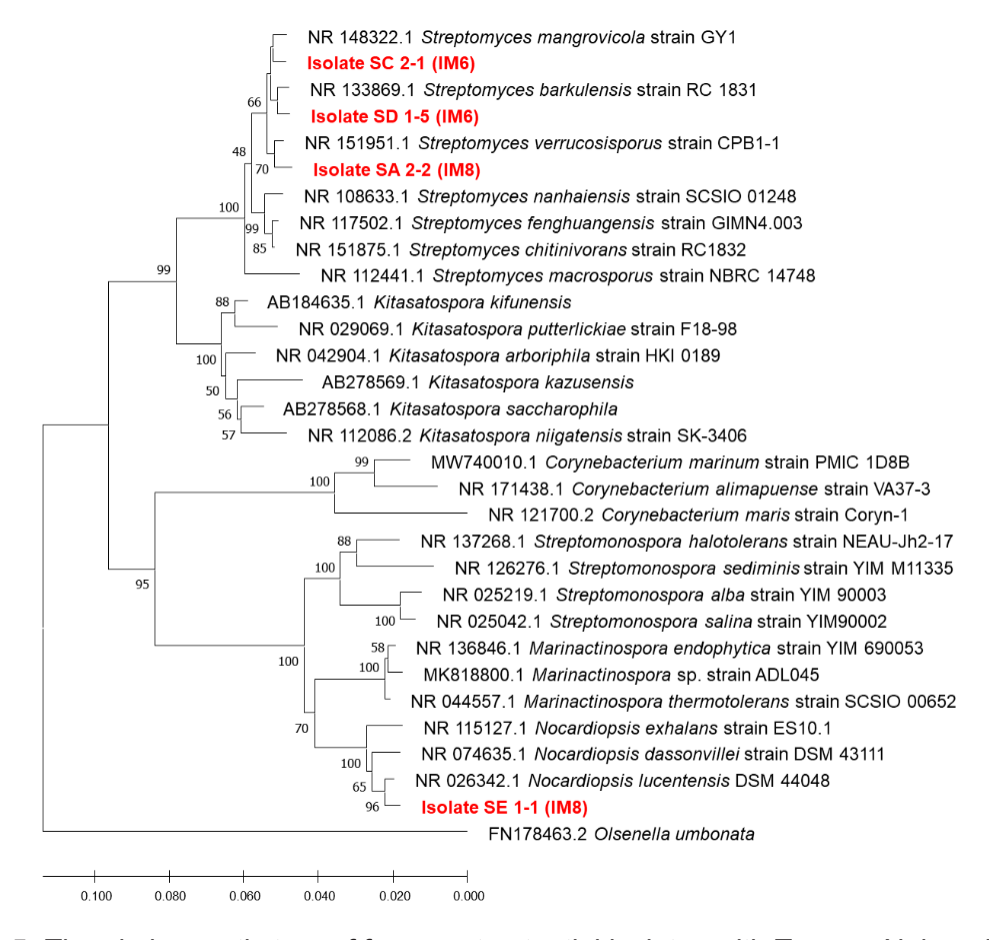
The phylogenetic tree of four most potential isolates with Tamura-Nei model and bootstrap 1000 constructed using MEGA X.

The phylogenetic position of isolates SA 2.2 (IM8), SC 2.1 (IM6), SD 1.5 (IM6) and SE 1.1 (IM8) in the phylogenetic tree can be seen in Fig. 5. Fig. 5 demonstrated that SA 2.2 (IM8), SC 2.1 (IM6) and SD 1.5 (IM6) were classified into the family *Streptomycetaceae* and SE 1.1 (IM8) was classified in *Nocardiopsis*. The family *Streptomycetacea* is represented by the genera *Streptomyces* and *Kitasatospora*. Moreover, family *Nocardiopsaceae* is represented by genera *Corynebacterium, Streptomonospora, Marinactinospora* and *Nocardiopsis*.

## DISCUSSION

Microbial protease and lipase are the hydrolytic enzymes with the highest consumption in a global market because of their important application in the industrial processes (Mukesh *et al*. 2012; Mahajan *et al*. 2016). Therefore, it is important to explore new sources of microbial protease producers, one of them from actinobacteria, since actinobacteria are well known as biologically active compounds producers, including enzymes. *L. vannamei* pond sediments are considered as underexplored sources of actinobacterial isolates. Most of actinobacteria are isolated from terrestrial and marine environment (Bernal *et al*. 2015; Babu *et al*. 2018). Beside that, vannamei ponds also have unique characteristics in the water salinity, since it is a mixture between freshwater from a river and saltwater from the sea. This unique characteristic affects the biodiversity of actinobacteria that live in the sediments. Only halotolerant actinobacteria that can survive in such kind of environment. In this study, the isolation of actinobacteria from pond sediments shows promising isolates in term of biodiversity and primary metabolite production. Amongst 40 isolates, all of them shows different character through morphological as well as microscopic observation. Furthermore, half of them are able to produce hydrolytic enzymes, which can be employed in the future for more sustainable aquaculture. Actinobacteria in aquaculture ecosystem play role as biocontrol agents, probiotic, bioremediation agent and many more (Balagurunathan *et al*. 2020; Shijila Rani *et al*. 2022).

The identification of actinobacteria in this study, reveal that pond sediments are dominated by the *Streptomyces* genus. Most of the isolates has powdery surface appearance, with aerial hyphae mostly gray or white and many spores produced. The microscopic observation also supports this finding, that most of the isolates have branching substrate and aerial hyphae with no septae and various spores shape (Appendix). Research from Klausen *et al*. (2005) also reported that genus *Streptomyces* is the dominant genus found in fish pond ecosystems. Another research from Manikkam *et. al*. (2020) is using metagenomic approach to estimate the abundance of actinobacteria from pond ecosystem, reported that *Streptomyces* are the second dominated genus with 14.64% then followed by *Nocardia* as many as 4.55%. *Strepptomyces* spp. are well studied genera that has significant roles in ecology as well as industry (Chevrette *et al*. 2019). Many protease and lipase producers were found in genus *Streptomyces*, such as *Streptomyces bambergiensis* (Ugur *et al*. 2014), *Streptomyces* sp. Al-Dhabi-49 (Al-Dhabi *et al*. 2020), *Streptomyces griseus* (Vishnupriya *et al*. 2010), *S. thermovulgaris* (De Azeredo *et al*. 2006), and *Streptomyces thermonitrificans*, *Streptomyces flavogriseus* HS1 (Ghorbel *et al*. 2014). *Streptomyces* and *Nocardiopsis* has been widely recognice for the production of enzymes that usefull for various industrial process (Sun *et al*. 2019; Al-Dhabi *et al*. 2019).

Beside genus *Streptomyces*, this study also found that genus *Nocardiopsis* has the potential to produce hydrolytic enzymes. *G*enus *Nocardiopsis* was first described by Meyer (1976) based on morphological characteristics and the chemical composition of cells. This genus member can be found in a wide range of areas, including alkaline slag dump, indoor environments, clinical material, and household waste, however, predominantly reported from alkaline or saline soils (Yassin *et al*. 2009; Akhwale *et al*. 2016). In support of our finding, another *Nocardiopsis* sp. was also found to be able to produce alkaline protease (Mohamedin 1999). Beside the industrial function, microbial enzymes from *Streptomyces* or *Nocardiopsis* are also able to degrade the organic waste in environment and can be used in waste water treatment as a bioremediation agent. Research from Patel *et al*. (2021) reported that *Nocardiopsis alba* is able to produce Triacylglycerol (EC 3.1.1.3) lipase, which can reduce the pollution load in wastewater of textile industry effluent, proofed by the reducing value of biochemical oxygen demand (BOD), chemical oxygen demand (COD), and total solids (TS).

Both genera of *Nocardiopsis* and *Streptomyces* belong to the phylum Actinobacteria. This Actinobacteria phylum contains six major classes: Acidimicrobiia, Actinobacteria, Coriobacteria, Nitriliruptoria, Rubrobacteria and Thermoleophilia (Ludwig *et al*. 2015). All of the species used to construct the phylogenetic tree are from the brackish or marine ecosystems. Class actinobacteria itself consist of 15 orders and one order *Incerta sedis* (Ludwig *et al*. 2015). The exploration of actinobacteria from vannamei pond sediment shows abundant predominated *Streptomyces* spp. live in it. Those actinobacteria might involve in biogeochemical cycle especially carbon cycle, proofed by the ability to produced hydrolytic enzymes, that function to breakdown organic matters become smaller molecules. Further biotechnological research is needed to examine the ability of those isolated actinobacteria in bioremediation process of *L. vannamei* ponds ecosystems.

## CONCLUSION

In conclusion, actinobacteria are promising source of hydrolytic enzyme producers especially protease and lipase. Among 40 isolates, 15 of them (37.5%) are able to show proteolytic activity, and 20 of them (50%) able to show lipolytic activity. The diversity of actinobacteria species that produce hydrolytic enzymes is dominated by the genus *Streptomyces*. Furthermore, the most potential isolates that performed high IP and LAI are *S. mangrovicola* SC 2.1, *S. barkulensis* SD 1.5, *S. verrucosisporus* SA 2.2 and *Nocardiopsis lucetensis* SE 1.1.

## Figures and Tables

**Figure 4:**
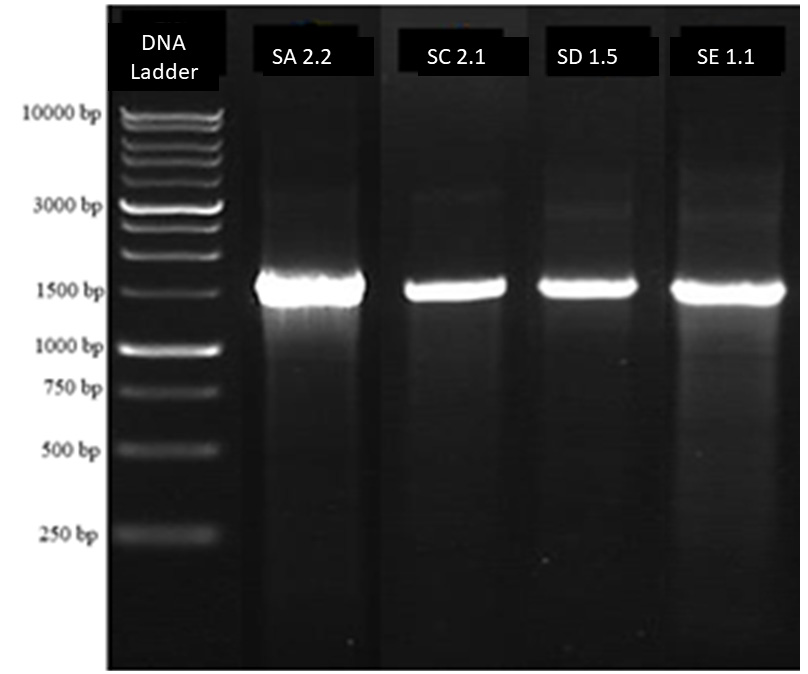
The UV-Doc result from four most potential isolates showing DNA band with length 1,500 bp.

**Table 1:** The proteolytic and lipolytic activity of actinobacteria.

No	Medium	Isolate code	Lipolytic Activity Index (LAI)	Index of Protease (IP)
1	IM8	SA 2.1	1.30	0.00
2	IM8	SA 2.2	6.50	0.00
3	IM8	SA 2.4	1.20	0.00
4	IM8	SA 2.5	1.42	0.00
5	IM8	SA 2.6	0.00	0.00
6	IM8	SA 2.7	0.00	1.60
7	IM6	SA 3.1	0.00	4.00
8	IM6	SA 4.1	0.00	0.00
9	IM8	SA 4.1	0.00	0.00
10	IM8	SA 4.2	0.00	0.00
11	IM8	SB 1.1	2.80	0.00
12	IM8	SB 1.2	6.40	0.00
13	IM6	SB 1.2	0.00	0.00
14	IM6	SB 1.3	2.40	0.00
15	IM6	SB 1.4	2.06	0.00
16	IM6	SB 1.5	1.70	0.00
17	IM6	SB 1.6	0.00	0.00
18	IM8	SB 1.6	0.00	0.00
19	IM8	SB 2.4	1.46	0.00
20	IM8	SB 2.6	1.84	0.00
21	IM6	SB 4.1	1.94	1.20
22	IM6	SB 4.2	1.62	0.00
23	IM6	SC 2.1	2.22	2.40
24	IM6	SC 2.2	2.20	0.00
25	IM6	SC 2.3	0.00	4.30
26	IM8	SC 3.1	2.12	1.40
27	IM6	SC 3.1	0.00	0.00
28	IM8	SC 3.2	1.97	0.00
29	IM6	SC 3.3	3.20	0.00
30	IM8	SC 3.3	1.87	3.13
31	IM8	SC 3.4	0.00	2.40
32	IM8	SD 1.1	0.00	1.60
33	IM8	SD 1.2	0.00	2.00
34	IM8	SD 1.3	0.00	1.70
35	IM8	SD 1.4	0.00	0.00
36	IM6	SD 1.5	0.00	3.50
37	IM8	SD 1.5	0.00	
38	IM8	SE 1.1	1.75	2.17
39	IM8	SE 1.3	0.00	2.61
40	IM8	TGSE 4	0.00	1.31

**Table 2:** BLAST homology result for the most promising isolates.

No.	Isolate code	Closest species	Length of basepair	Per indent	Reference no.	Accession no.
1	SA 2.2 (IM8)	*Streptomyces verrucosisporus*	1,431	99.36%	NR_151951.1	LC655154
2	SC 2.1 (IM6)	*Streptomyces mangrovicola*	1,429	99.15%	NR_148322.1	LC655157
3	SD 1.5 (IM6)	*Streptomyces barkulensis*	1,429	99.21%	NR_133869.1	LC655153
4	SE 1.1 (IM8)	*Nocardiopsis lucetensis*	1,430	99.23%	NR_026342.1	LC655155

## APPENDIX

**Table:** Supplementary Material Observation of actinobacterial colony and microscopy
